# Estetrol/drospirenone for combined oral contraception: a systematic review of efficacy, cycle control, and safety

**DOI:** 10.3389/fendo.2026.1867343

**Published:** 2026-07-24

**Authors:** Manuel Sánchez-Prieto, Sandra Coll, Marina Romero-Domínguez, Marta Avella-Marcos, Mireia Castilla, Margarita Gómez del Valle, Silvia Franco-Camps

**Affiliations:** 1 Department of Obstetrics and Gynecology, Dexeus Mujer, Hospital Universitario General de Catalunya, Sant Cugat del Vallés, Spain; 2 Department of Obstetrics and Gynecology, Hospital QuironSalud Málaga, Málaga, Spain

**Keywords:** combined oral contraceptives, cycle control, drospirenone, estetrol, grade, hormonal contraception, safety, systematic review

## Abstract

**Objective:**

To systematically evaluate the evidence on the contraceptive efficacy, cycle control, safety, and selected noncontraceptive effects of estetrol 15 mg/drospirenone 3 mg (E4/DRSP) and to assess certainty of evidence by outcome using the GRADE approach.

**Methods:**

A systematic review was conducted in accordance with PRISMA 2020. PubMed/MEDLINE, Scopus, and Google Scholar were searched for eligible studies published through February 2026. Clinical trials and comparative studies evaluating E4/DRSP in women of reproductive age were included. Outcomes were grouped into contraceptive efficacy, bleeding/cycle control, safety and tolerability, hemostatic and endocrine–metabolic effects, ovarian suppression, and noncontraceptive clinical outcomes. Risk of bias was assessed using design-specific tools, and certainty of evidence was graded by outcome.

**Results:**

A total of 25 eligible publications/study reports were included, comprising phase 2 dose-finding studies, pivotal phase 3 contraceptive trials, pooled analyses, mechanistic comparator studies, adolescent data, and indication-specific studies. E4/DRSP demonstrated robust contraceptive efficacy, predictable bleeding patterns, and an acceptable tolerability profile. Across mechanistic studies, E4/DRSP showed less pronounced hemostatic and endocrine–metabolic effects than ethinyl estradiol–containing comparators. The most consistent biological differentiation of E4/DRSP was observed for APC resistance and thrombin-generation endpoints, which were less affected than with EE-containing comparators. The strongest noncontraceptive evidence was observed for dysmenorrhea, supported by a randomized, double-blind, placebo-controlled trial. Certainty of evidence was moderate for contraceptive efficacy, cycle control, common adverse events, and surrogate hemostatic/metabolic outcomes; high for dysmenorrhea versus placebo; low for endometriosis-related and menstrual symptom outcomes; and very low for clinical thromboembolic risk.

**Conclusions:**

E4/DRSP is an effective combined oral contraceptive with favorable cycle control and a consistent biologic profile suggesting lower hepatic/hemostatic impact than ethinyl estradiol–containing formulations. However, current evidence does not establish comparative thromboembolic safety, which requires dedicated postauthorization and real-world comparative studies.

## Introduction

Combined oral contraceptives (COCs) remain one of the most widely used reversible contraceptive methods worldwide because of their high contraceptive effectiveness, cycle regulation, and additional noncontraceptive benefits (1–5). Despite decades of clinical use and continuous reformulation, the balance between efficacy, tolerability, bleeding control, and safety remains a central issue in contraceptive development (2, 3). Estrogen-related hepatic effects, hemostatic changes, unscheduled bleeding, and treatment discontinuation continue to shape both prescribing decisions and user satisfaction (2, 3). These considerations are especially relevant in contemporary contraceptive care, where individualized method selection increasingly emphasizes not only contraceptive efficacy, but also metabolic profile, menstrual acceptability, and overall tolerability (1–3).

Most currently available COCs contain ethinyl estradiol (EE), a synthetic estrogen with potent hepatic activity that contributes substantially to contraceptive efficacy but also to changes in coagulation, fibrinolysis, lipid metabolism, angiotensinogen, and sex hormone-binding globulin (SHBG) (3). Although modern EE-containing formulations are generally safe in appropriately selected users, their biologic effects continue to motivate the search for estrogen components with a more favorable pharmacologic profile (3). Estetrol (E4) is a native fetal estrogen with distinctive tissue-selective activity and lower hepatic impact than EE in preclinical and clinical pharmacology studies (4, 6–8). When combined with drospirenone (DRSP), a progestin with anti-mineralocorticoid and anti-androgenic properties, E4 has been developed as a novel COC intended to preserve contraceptive efficacy while potentially reducing estrogen-related metabolic and hemostatic perturbation (8).

Clinical development programs of E4–15 mg/DRSP 3 mg have evaluated multiple domains relevant to contraceptive practice, including contraceptive efficacy, scheduled and unscheduled bleeding patterns, tolerability, cardiovascular and thromboembolic safety signals, ovarian suppression, and noncontraceptive outcomes such as dysmenorrhea, endometriosis-associated pain, and premenstrual or menstrual symptom burden (6–8). Phase 3 studies have reported low pregnancy rates, predictable bleeding patterns, and acceptable discontinuation rates, while mechanistic comparative studies have suggested less pronounced effects on thrombin generation, activated protein C resistance, SHBG, triglycerides, and related biomarkers than EE-containing comparators (9–11). At the same time, the available literature is heterogeneous in design and clinical purpose, encompassing pivotal trials, pooled analyses, open-label studies, active-comparator physiologic studies, and *post hoc* analyses (11).

This heterogeneity creates an important interpretive challenge. Favorable changes in surrogate hemostatic or metabolic markers do not necessarily translate into definitive reductions in clinical events such as venous thromboembolism, particularly when available trials are not powered for rare outcomes. Evidence supporting noncontraceptive benefits varies substantially by indication, comparator, and study design (3, 4). A rigorous synthesis requires not only narrative integration of the available evidence, but also structured appraisal of certainty by outcome. In this context, the GRADE framework is particularly valuable because it allows separate assessment of the confidence that can be placed in estimates for contraceptive efficacy, bleeding control, tolerability, thromboembolic safety, and noncontraceptive benefits (12).

We conducted a systematic review of the published evidence on E4–15 mg/DRSP 3 mg as a combined oral contraceptive, with PRISMA-oriented study identification and selection, and outcome-level certainty assessment using GRADE. Our objective was to synthesize the evidence on contraceptive efficacy, cycle control, safety, and selected noncontraceptive effects, while distinguishing more robust findings from those supported primarily by mechanistic or lower-certainty data.

## Methods

### Study design and reporting framework

We conducted a systematic review of the published literature on estetrol 15 mg/drospirenone 3 mg (E4/DRSP) as a combined oral contraceptive. The review was designed and reported in accordance with the Preferred Reporting Items for Systematic Reviews and Meta-Analyses (PRISMA) 2020 statement. The protocol was not prospectively registered, due to the exploratory and heterogeneous nature of the available evidence base. Eligibility criteria and the review framework were defined *a priori*. Given the heterogeneity of the available evidence, including clinical development studies, mechanistic comparator trials, pooled analyses, and *post hoc* reports, a qualitative systematic synthesis was performed, with outcome-level certainty of evidence assessed using the GRADE approach rather than quantitative meta-analysis.

### Information sources and search strategy

A structured search was performed in PubMed/MEDLINE, Scopus, and Google Scholar for articles published through February 2026. The core search terms were “estetrol”, “drospirenone”, and “contraception”, used alone and in combination. The reference lists of relevant publications were also screened manually to identify additional eligible reports. Only full-text publications reporting original human data were considered.

Additional postmarketing pharmacovigilance, predictive-modeling, and registry sources published or updated after the formal search date were cited only as contextual evidence in the Discussion and were not included in the formal synthesis or GRADE assessment.

The full electronic search strategies for all databases are provided in **Supplementary Material 1**.

### Eligibility criteria

Eligible reports met prespecified criteria defined according to the PICO framework. Studies were included if they:

1. evaluated E4/DRSP, preferably the marketed 15 mg/3 mg 24/4 regimen;
2. enrolled women of reproductive age or adolescent users in clinically relevant contraceptive settings;
3. reported at least one outcome related to contraceptive efficacy, bleeding/cycle control, safety, tolerability, ovarian suppression, hemostatic or endocrine-metabolic parameters, or noncontraceptive clinical outcomes; and
4. used an interventional or comparative design, including randomized clinical trials, nonrandomized comparative studies, pooled analyses from clinical trials, and prespecified or *post hoc* analyses derived from eligible studies.

We excluded narrative reviews, expert opinions, editorials, letters without original data, conference abstracts lacking sufficient methodological detail, and duplicate publications that did not contribute additional outcome data. Early dose-finding studies that included nonmarketed E4 regimens were retained only when they provided supportive mechanistic or pharmacodynamic information relevant to interpretation of the approved E4/DRSP formulation and were analyzed separately from pivotal contraceptive efficacy studies. This distinction was considered especially important because the available evidence spans efficacy studies, bleeding-pattern analyses, cardiovascular safety analyses, mechanistic hemostatic studies, endocrine-metabolic comparator trials, and indication-specific studies in dysmenorrhea and endometriosis.

### PICO framework

The review question was structured according to a PICO framework (Population, Intervention, Comparator, Outcomes), as summarized in **Table 1**.

**Table 1:** PICO framework of the systematic review.

Component	Definition in this review
Population (P)	Women of reproductive age and adolescent users in clinically relevant contraceptive settings, including general contraceptive populations and selected subgroups (e.g., dysmenorrhea, endometriosis, PCOS)
Intervention (I)	Estetrol 15 mg/drospirenone 3 mg (E4/DRSP), preferably the marketed 24/4 regimen
Comparator (C)	No comparator (single-arm phase 3 trials), placebo, or active comparators including ethinyl estradiol–containing combined oral contraceptives
Outcomes (O)	Contraceptive efficacy, bleeding/cycle control, safety and tolerability, venous thromboembolism, hemostatic and endocrine–metabolic parameters, ovarian suppression, and noncontraceptive outcomes (dysmenorrhea, endometriosis-related pain, menstrual symptoms)

The review question was structured according to the Population, Intervention, Comparator, and Outcomes (PICO) framework. This structure guided eligibility criteria, study selection, and outcome classification across heterogeneous study designs, including interventional trials, pooled analyses, mechanistic comparator studies, and *post hoc* reports.

### Study selection

Records identified through database searching and manual screening were imported into a structured database for screening and data management.

Two reviewers independently screened titles and abstracts to exclude records that were clearly irrelevant to the review question. Full-text articles were subsequently assessed for eligibility according to predefined inclusion and exclusion criteria based on the PICO framework.

Discrepancies in study selection were resolved through discussion and consensus. No automation tools were used in the screening process.

### Data extraction

Two reviewers independently extracted data using a standardized form. The following variables were collected from each eligible report: first author, year of publication, geographic setting, study period when available, sample size, population characteristics, study design, comparator, treatment duration or follow-up, objective, and main outcomes. Outcomes were grouped *a priori* into the following clinical domains:

1. contraceptive efficacy (pregnancy rate, Pearl Index, life-table estimates);
2. bleeding and cycle control (scheduled bleeding, unscheduled bleeding/spotting, bleeding-related discontinuation);
3. safety and tolerability (treatment-emergent adverse events, serious adverse events, discontinuation due to adverse events, cardiovascular complaints, venous thromboembolism);
4. physiologic and mechanistic endpoints (ovarian suppression, thrombin generation, activated protein C resistance, fibrinolytic markers, SHBG, lipids, and related endocrine-metabolic markers); and
5. noncontraceptive clinical outcomes (dysmenorrhea, endometriosis-associated pain, adenomyosis-related symptoms, and premenstrual/menstrual symptom burden). Discrepancies in extraction were resolved by consensus.

### Risk-of-bias assessment

Risk of bias was assessed at the study level using design-specific tools. Randomized trials were evaluated with the revised Cochrane Risk of Bias tool for randomized trials (RoB 2), whereas nonrandomized comparative studies were assessed according to ROBINS-I principles. For single-arm, pooled, *post hoc*, and subgroup analyses, a design-adapted assessment was applied, focusing on participant selection, exposure definition, outcome measurement, completeness of outcome data, selective reporting, overlapping study populations, and sponsorship-related concerns. Two reviewers independently performed the assessments, with disagreements resolved by consensus. Reporting bias/publication bias was not formally assessed because no quantitative meta-analysis was performed and the evidence base was heterogeneous in design, outcome definitions, comparators, and populations.

### Certainty of the evidence

The certainty of evidence was evaluated by outcome using the Grading of Recommendations Assessment, Development and Evaluation (GRADE) framework (12). Certainty judgments considered risk of bias, inconsistency, indirectness, imprecision, and publication bias, and were made separately for each major outcome domain rather than globally for the product. Caution was applied when interpreting surrogate hemostatic and metabolic markers, because favorable biologic profiles do not necessarily establish reductions in rare clinical outcomes such as venous thromboembolism. Similarly, *post hoc* analyses and symptom-based open-label studies were considered more vulnerable to bias and indirectness than blinded randomized trials with patient-centered endpoints. Because multiple reports arose from the same phase 3 program, GRADE ratings were based on the overall body of evidence for each outcome, not on simple article counts.

### Data synthesis

Because of substantial heterogeneity in design, comparator, follow-up duration, population, and endpoint definition, a pooled meta-analysis was not considered appropriate. Evidence was synthesized narratively and organized by prespecified outcome domain: contraceptive efficacy, cycle control, safety and tolerability, hemostatic and endocrine-metabolic effects, and noncontraceptive clinical outcomes. When available, absolute event rates, effect estimates, and confidence intervals reported by the original studies were extracted and summarized descriptively. Mechanistic studies evaluating coagulation, fibrinolysis, thrombin generation, SHBG, or metabolic parameters were interpreted as supportive biologic evidence and were not used as substitutes for clinical vascular safety outcomes. Findings from *post hoc* or subgroup analyses were interpreted as complementary rather than confirmatory evidence.

An evidence map was constructed to visually integrate outcome domains, certainty of evidence, and characteristics of the contributing evidence base.

Because no quantitative meta-analysis was performed and the included evidence was heterogeneous in design, outcome definition, and population, with partial overlap across related study reports, formal assessment of reporting bias/publication bias was not undertaken.

### Presentation of results

**Figure 1:**
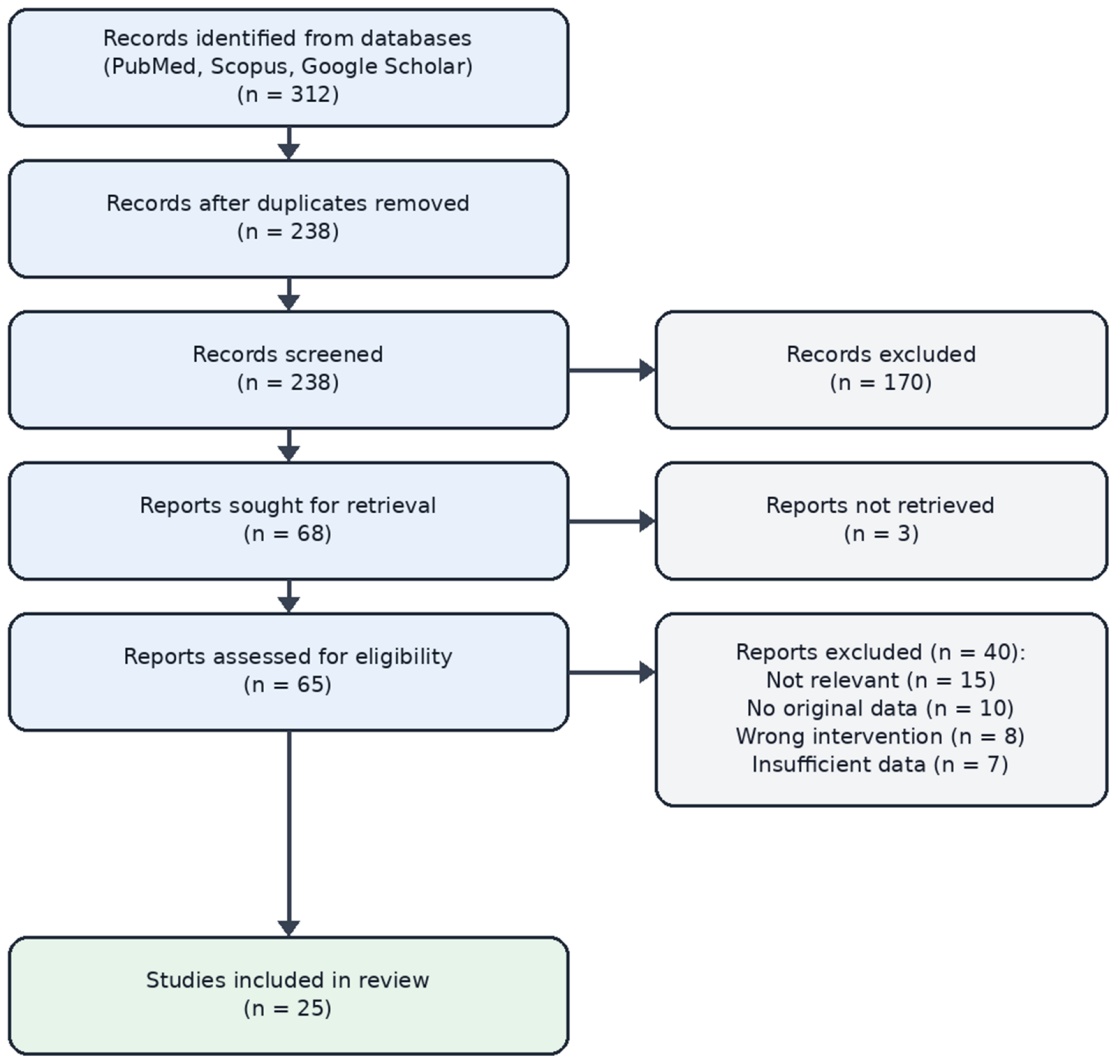
PRISMA 2020 flow diagram of study selection.

**Figure 2:**
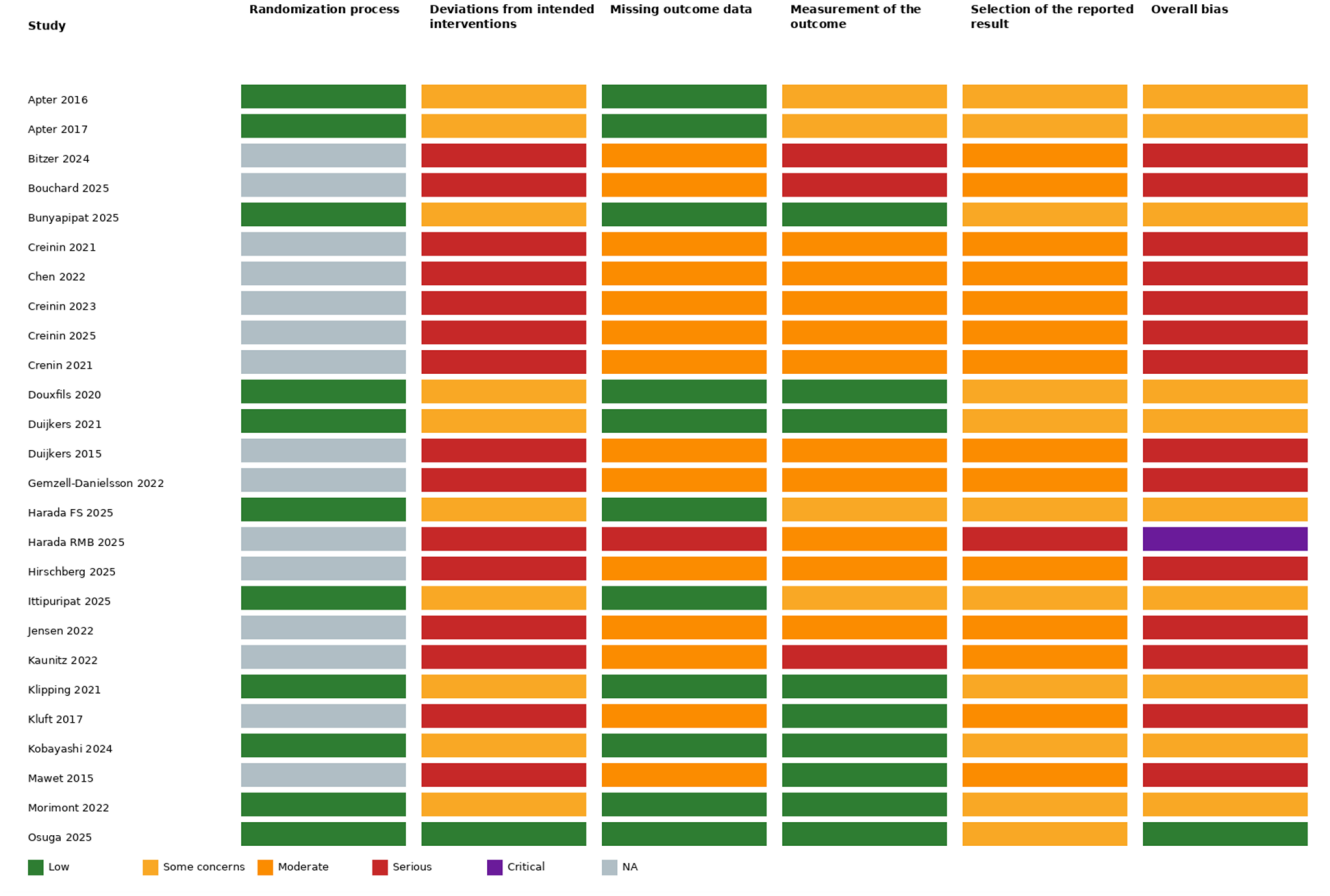
Risk-of-bias assessment of included publications/study reports. (A) Study-level risk-of-bias judgments across domains. (B) Summary of risk-of-bias judgments presented as proportions across domains, based on 25 included publications/study reports. Percentages were calculated directly from the finalized study-level risk-of-bias table. Randomized trials were assessed using RoB 2, whereas nonrandomized, pooled, single-arm, and *post hoc* reports were evaluated using design-adapted criteria informed by ROBINS-I principles. Several publications originated from the same phase 3 clinical development program and were considered related study reports rather than independent study populations.

Study selection is presented in a PRISMA 2020 flow diagram (**Figure 1**). Study characteristics are summarized in **Table 2**, distinguishing pivotal contraceptive efficacy studies, pooled or secondary analyses from phase 3 programs, mechanistic comparator trials, adolescent studies, and indication-specific noncontraceptive studies. Risk-of-bias assessments are presented in **Figure 2** and were incorporated into the outcome-level GRADE judgments summarized in **Table 3**.

**Table 2:** Characteristics of included studies evaluating estetrol 15 mg/drospirenone 3 mg (E4/DRSP).

Study (year)	Country/region	Study design	Population	Sample size (n)	Comparator	Duration	Primary objective	Key outcomes domain
Pivotal contraceptive efficacy trials								
Creinin (2021) (9)	USA/Canada	Phase 3, multicenter, open-label	Women 16–35 y	1864	None	13 cycles	Contraceptive efficacy, bleeding, safety	Efficacy, bleeding, safety
Gemzell-Danielsson (2022) (7)	Europe/Russia	Phase 3, multicenter, open-label	Women 18–50 y	1553	None	12 cycles	Efficacy, bleeding pattern, safety	Efficacy, bleeding, safety
Jensen (2022) (13)	USA	Phase 3 analysis	Subgroup analysis	3027	None	Not stated	Efficacy across subgroups	Efficacy
Creinin (2023) (14)	USA	Phase 3 secondary analysis	Phase 3 cohort	2837	None	Not stated	Adherence and pregnancy risk	Efficacy
Bleeding and cycle control analyses								
Kaunitz (2022) (15)	USA	Phase 3 analysis	Phase 3 cohort	3409	None	Not stated	Bleeding pattern	Bleeding
Apter (2016) (16)	Finland	Randomized, open-label, dose-finding	Healthy women	396	E4/LNG	Cycles	Bleeding profile	Bleeding
Apter (2017) (17)	Finland	Randomized trial	Healthy women	396	E4/LNG	Cycles	Acceptability, weight, well-being	Tolerability
Hirschberg (2025) (18)	Sweden	Phase 3, open-label	Adolescents	105	None	6 cycles	Safety and bleeding	Bleeding, safety
Safety and pooled analyses								
Chen (2022) (19)	USA	Pooled phase 3 analysis	Phase 3 participants	3725	None	Not stated	Safety and tolerability	Safety
Creinin (2025) (10)	USA	Phase 3 pooled analysis	Women with CV risk factors	3417	None	Not stated	Cardiovascular safety	Safety
Mechanistic and comparative studies (hemostasis/metabolism)								
Douxfils (2020) (20)	Netherlands	Randomized controlled trial	Healthy women	101	EE/LNG, EE/DRSP	6 cycles	Hemostatic effects	Hemostasis
Morimont (2022) (21)	Belgium	Randomized controlled trial	Healthy women	86	EE/LNG, EE/DRSP	6 cycles	Thrombin generation	Hemostasis
Kobayashi (2024) (22)	Japan	Randomized, open-label	Endometriosis patients	82	EE/DRSP	Not stated	Coagulation/fibrinolysis	Hemostasis
Klipping (2021) (23)	Netherlands	Randomized, open-label	Healthy women	98	EE/LNG, EE/DRSP	6 cycles	Endocrine/metabolic effects	Metabolic
Kluft (2017) (24)	Netherlands	Open-label, dose-finding	Healthy women	47	EE/DRSP	Not stated	Hemostasis markers	Hemostasis
Mawet (2015) (25)	Netherlands	Controlled, dose-finding	Healthy women	109	EE/DRSP	Not stated	Metabolic and hepatic safety	Metabolic
Bunyapipat (2025) (26)	Thailand	Randomized crossover	PCOS women	61	EE/DRSP	Not stated	Glucose metabolism	Metabolic
Ovarian suppression and physiology								
Duijkers (2021) (27)	Netherlands	Randomized, open-label	Healthy women	82	EE/DRSP	3 cycles	Ovarian suppression	Physiology
Duijkers (2015) (28)	Netherlands	Phase 2 dose-finding	Healthy women	109	E4 regimens	Not stated	Ovulation inhibition	Physiology
Ittipuripat (2025) (29)	Thailand	Randomized, single-blind	Healthy women	36	EE/GS	Not stated	Delayed-start efficacy	Physiology
Noncontraceptive outcomes								
Osuga (2025) (30)	Japan	RCT, double-blind, placebo-controlled	Dysmenorrhea	162	Placebo	16 weeks	Pain reduction	Dysmenorrhea
Harada (2026) (31)	Japan	*Post hoc* analysis	Adenomyosis subgroup	162	Placebo	16 weeks	Dysmenorrhea in adenomyosis	Dysmenorrhea
Harada (2025) (32)	Japan	Randomized, open-label	Endometriosis	88	EE/DRSP	12 weeks	Pelvic pain	Endometriosis
Bitzer (2024) (33)	Europe	Phase 3, open-label	Women	1398	None	Not stated	Premenstrual symptoms	Symptoms
Bouchard (2025) (34)	North America	Phase 3, open-label	Women	1308	None	Not stated	Premenstrual symptoms	Symptoms

Studies are grouped according to their primary evidence domain. Several publications originated from the same phase 3 clinical development program and are presented as related study reports rather than independent study populations. E4, estetrol; DRSP, drospirenone; EE, ethinyl estradiol; LNG, levonorgestrel; GS, gestodene; PCOS, polycystic ovary syndrome; RCT, randomized controlled trial.

**Table 3:** Certainty of evidence (GRADE) by outcome.

Outcome	Participants (approx.)	Study design	Risk of bias	Inconsistency	Indirectness	Imprecision	Publication bias	Certainty
Contraceptive efficacy	~7,000	Phase 3 open-label trials + secondary analyses	Serious	Not serious	Not serious	Not serious	Not formally assessed	⊕⊕⊕◯ Moderate
Cycle control/bleeding	~5,000	Phase 3 open-label trials	Serious	Not serious	Not serious	Not serious	Not formally assessed	⊕⊕⊕◯ Moderate
Common adverse events	~7,000	Phase 3 + pooled analyses	Serious	Not serious	Not serious	Not serious	Not formally assessed	⊕⊕⊕◯ Moderate
Venous thromboembolism	~7,000	Phase 3 + pooled analyses	Serious	Not serious	Serious	Very serious	Not formally assessed	⊕◯◯◯ Very low
Hemostatic markers	~250	Randomized comparator studies	Not serious	Not serious	Serious	Serious	Not formally assessed	⊕⊕⊕◯ Moderate
Endocrine–metabolic markers	~200	Randomized comparator studies	Not serious	Not serious	Serious	Serious	Not formally assessed	⊕⊕⊕◯ Moderate
Dysmenorrhea (vs placebo)	162	Randomized double-blind trial	Not serious	Not serious	Not serious	Not serious	Not formally assessed	⊕⊕⊕⊕ High
Endometriosis-related pain	~88	Open-label randomized study	Serious	Not serious	Serious	Serious	Not formally assessed	⊕⊕◯◯ Low
Menstrual/premenstrual symptoms	~2,700	Phase 3 open-label analyses	Serious	Not serious	Serious	Not serious	Not formally assessed	⊕⊕◯◯ Low

Certainty of evidence was assessed using the GRADE approach and is presented by outcome domain. Judgments were based on risk of bias, inconsistency, indirectness, imprecision, and publication bias. Evidence was derived from phase 2–3 clinical trials, pooled analyses, mechanistic comparator studies, and *post hoc* reports. Several publications originated from the same clinical development program and were considered related study reports rather than independent study populations. Publication bias was not formally assessed due to the absence of quantitative meta-analysis and the heterogeneity and partial overlap of included study reports.

E4, estetrol; DRSP, drospirenone; RCT, randomized controlled trial; VTE, venous thromboembolism.

## Results

### Study selection

The study selection process is summarized in **Figure 1**.

Reports from the same phase 3 clinical development program were considered related study reports rather than independent study populations.

A total of 312 records were identified through database searching (PubMed, Scopus, and Google Scholar). After removal of 74 duplicate records, 238 records were screened based on title and abstract, of which 170 were excluded.

A total of 68 reports were sought for retrieval, of which 3 were not retrieved. The remaining 65 reports were assessed for eligibility in full text. Following full-text review, 40 reports were excluded for predefined reasons, including lack of relevant clinical data, absence of original data, evaluation of non-relevant interventions, or insufficient methodological detail.

Finally, 25 eligible publications/study reports were included in the qualitative synthesis. Reports arising from the same clinical development program were considered related study reports rather than independent study populations.

### Study characteristics

The characteristics of the included studies are summarized in **Table 2**.

A total of 25 eligible publications/study reports were included, comprising pivotal phase 3 contraceptive trials, pooled and secondary analyses derived from the phase 3 development program, mechanistic comparator studies, adolescent data, and indication-specific studies evaluating noncontraceptive outcomes.

The evidence base was dominated by the phase 3 clinical development program, which provided the primary data on contraceptive efficacy, bleeding patterns, and safety, while mechanistic studies contributed data on hemostatic and endocrine–metabolic effects, and smaller studies addressed ovarian function and noncontraceptive outcomes.

### Risk of bias across included reports

Risk-of-bias assessments across included publications/study reports are summarized in **Figure 2**. Overall, the risk of bias varied across study designs and outcome domains, reflecting the heterogeneity of the evidence base.

For randomized controlled trials, including placebo-controlled and active-comparator studies, the risk of bias was generally low to some concerns, primarily related to the absence of blinding in open-label designs and, in some cases, limited reporting of allocation procedures or prespecified analyses. The double-blind, placebo-controlled trial evaluating dysmenorrhea showed the lowest overall risk-of-bias profile among the included studies (30).

The pivotal phase 3 contraceptive trials and related secondary analyses, which constitute the core evidence for efficacy, bleeding patterns, and safety, were predominantly open-label and single-arm. Accordingly, these studies were judged to have serious risk of bias in specific domains, particularly deviations from intended interventions and outcome measurement, driven by reliance on participant-reported adherence, bleeding patterns, and symptom outcomes. This limitation was less relevant for objective endpoints such as pregnancy.

Pooled analyses, subgroup analyses, and *post hoc* reports were also associated with serious to critical risk of bias, mainly due to the potential for selective reporting and the absence of independent control groups.

Mechanistic and physiologic studies, including those evaluating hemostatic and endocrine–metabolic parameters, showed low to some concerns in randomized comparator designs but higher risk of bias in noncomparative or dose-finding studies, in addition to inherent indirectness due to reliance on surrogate biomarkers.

Consistent with the distribution of judgments shown in **Figure 2B**, the main drivers of bias across the evidence base were open-label study design, reliance on patient-reported outcomes, limited sample size in mechanistic studies, and the inclusion of secondary or *post hoc* analyses. These limitations were explicitly incorporated into the outcome-level GRADE assessments, particularly for bleeding outcomes, symptom-based endpoints, and thromboembolic safety.

### Contraceptive efficacy

Contraceptive efficacy was supported primarily by large phase 3 studies and related secondary analyses. In the North American phase 3 trial including 1,864 women, the Pearl Index among women aged 16–35 years was 2.65 (95% CI, 1.73–3.88), with a method-failure Pearl Index of 1.43 (95% CI, 0.70–2.39) and a 13-cycle life-table pregnancy rate of 2.1%. In the Europe/Russia phase 3 study including 1,553 women, the pregnancy rate was 0.47 pregnancies per 100 woman-years (95% CI, 0.15–1.11), and the method-failure rate was 0.29 pregnancies per 100 woman-years (95% CI, 0.06–0.83). A large subgroup analysis including 3,027 participants found that E4/DRSP remained effective across age, body mass index, and contraceptive-history strata. A related adherence analysis showed that pregnancy risk increased with missed active pills and exceeded 1% only when more than two hormone-containing pills were missed within a 28-day cycle; among participants reporting complete pill use, the estimated pregnancy risk was 0.09% per cycle.

### Bleeding profile and cycle control

Across dose-finding, pivotal phase 3, and secondary bleeding analyses, E4/DRSP showed a generally favorable bleeding profile with predictable scheduled bleeding and declining unscheduled bleeding over time. In the North American phase 3 study, scheduled bleeding occurred in 82.9% to 87.0% of women per cycle, with a median duration of 4.5 days; unscheduled bleeding decreased from 30.3% in cycle 1 to 21.3%–22.1% in cycles 2–4, then stabilized at 15.5%–19.2% thereafter. In the Europe/Russia phase 3 study, scheduled bleeding/spotting occurred in 91.9%–94.4% of women during cycles 1–12, with a median duration of 4–5 days per cycle, whereas the proportion with unscheduled bleeding/spotting fell from 23.5% in cycle 1 to <16% from cycle 6 onward. The phase 3 North American bleeding-pattern analysis similarly described a predictable bleeding profile with limited unscheduled bleeding, although both adherence and body mass index influenced bleeding patterns. Earlier dose-finding data also identified the 15 mg E4/3 mg DRSP regimen as the most favorable among the regimens tested. In adolescents, unscheduled bleeding/spotting decreased from 45.8% in cycle 1 to 14.5% in cycle 5, and the number of days with unscheduled bleeding/spotting declined from 9 to 6 days per cycle.

### Safety and tolerability

The overall tolerability profile of E4/DRSP was favorable and was characterized mainly by common adverse events typical of combined oral contraceptives. In the North American phase 3 trial, the most frequent adverse events were headache (5.0%) and metrorrhagia (4.6%), and 7.1% of women discontinued treatment because of an adverse event, most commonly metrorrhagia or menorrhagia. In the Europe/Russia phase 3 study, the most common adverse events were headache (7.7%), metrorrhagia (5.5%), vaginal hemorrhage (4.8%), and acne (4.2%), with 9.1% discontinuing because of treatment-related adverse events. The pooled phase 3 safety analysis, which included 3,725 participants, found that most reported adverse events were mild or moderate; 28.7% experienced at least one treatment-related adverse event, most commonly bleeding complaints (9.5%), breast pain/tenderness (4.0%), acne (3.3%), and mood disorder (3.2%). Treatment-related discontinuation occurred in 8.0%, with bleeding complaints (2.8%) and mood disorder (1.1%) being the only categories exceeding 1%. No clinically relevant changes in weight, blood pressure, heart rate, or routine laboratory parameters were reported in the pooled phase 3 analysis. In adolescents, no serious treatment-related adverse events or major safety concerns were observed.

### Venous thromboembolism and cardiovascular safety

Clinical thromboembolic events were rare across the contraceptive development program, but the available evidence was insufficient to estimate comparative vascular risk with precision. No venous thromboembolic events were reported in the North American phase 3 efficacy trial, whereas one treatment-related lower-extremity venous thromboembolism was reported in the Europe/Russia phase 3 study. In the pooled phase 3 safety analysis, three serious treatment-related adverse events were reported, including one venous thromboembolism, one worsening depression, and one ectopic pregnancy. In the cardiovascular-risk subgroup analysis of 3,417 participants, 41.3% had at least one cardiovascular risk factor and 9.0% had at least two; only six participants (0.18%) discontinued because of a cardiovascular complaint, including three cases of hypertension (0.09%) and one venous thrombosis. Among women with baseline blood pressure of at least 130/85 mmHg, hypertension-related discontinuation remained uncommon.

### Hemostatic and endocrine-metabolic effects

Mechanistic comparator studies consistently showed that E4/DRSP had a less pronounced effect on hemostatic and endocrine-metabolic markers than EE-containing formulations. In a randomized trial comparing E4/DRSP with EE/levonorgestrel and EE/DRSP, the median change in activated protein C resistance-based endogenous thrombin potential at cycle 6 was +30% with E4/DRSP versus +165% with EE/levonorgestrel and +219% with EE/DRSP. Changes in prothrombin fragment 1 + 2 and SHBG were also smaller with E4/DRSP. Another randomized study found that after six cycles, mean thrombin generation remained within the reference range in the E4/DRSP arm, whereas the EE-containing arms exceeded the upper reference threshold; all thrombin-generation parameters were significantly less affected by E4/DRSP. In Japanese women with endometriosis, coagulation and fibrinolysis were less altered with E4/DRSP than with EE/DRSP, with an approximately fourfold greater increase in activated protein C sensitivity ratio and a 4.7-fold higher D-dimer level in the EE/DRSP group. Endocrine-metabolic studies likewise showed more limited changes in gonadotropins, cortisol-binding globulin, angiotensinogen, SHBG, and triglycerides with E4/DRSP than with EE-containing comparators. In women with polycystic ovary syndrome, E4/DRSP was not associated with significantly different changes in 2-hour oral glucose tolerance, 1-hour insulin, or HOMA-IR compared with EE/DRSP in a randomized crossover trial.

### Ovarian suppression and return of ovulation

Physiologic studies supported effective suppression of ovulation with E4/DRSP during standard use. In a randomized comparative study, no participants ovulated while receiving E4/DRSP, whereas ovulation occurred in two participants receiving EE/DRSP. Both treatments comparably suppressed estradiol, progesterone, and endometrial thickness, although E4/DRSP exerted less suppression of follicle-stimulating hormone and luteinizing hormone. Return of ovulation occurred at a mean of 15.5 days after discontinuation of E4/DRSP. Earlier phase 2 dose-finding data also showed complete suppression of ovulation across treatment groups, with dose-related ovarian suppression and post-treatment ovulation recovery between 17 and 21 days after the last active treatment. By contrast, in a delayed-start noninferiority trial initiated on cycle days 7–9, ovulation inhibition was 61.11% in both groups, and the high ovulation rate precluded confirmation of noninferiority despite similar outcomes between E4/DRSP and EE/gestodene.

### Noncontraceptive clinical outcomes

Evidence for noncontraceptive benefits was strongest for dysmenorrhea. In a randomized, double-blind, placebo-controlled trial including 162 Japanese women with primary or secondary dysmenorrhea, E4/DRSP reduced the most severe total dysmenorrhea score by 2.3 points from baseline at week 16, with a significant between-group difference of −1.4 (95% CI, −1.8 to −1.0) (30). The responder rate, defined as a reduction of at least 2.0 points, was 64.3% with E4/DRSP versus 28.4% with placebo. Pelvic pain scores, dysmenorrhea symptoms during menstrual bleeding, quality-of-life measures, and global impression scores also improved. In a *post hoc* adenomyosis analysis derived from the same study population, the between-group difference in dysmenorrhea score was −1.8 (95% CI, −2.5 to −1.0), and the responder rate was 66.7% with E4/DRSP versus 20.0% with placebo (31). In endometriosis-associated pelvic pain, an open-label active-comparator trial found similar changes in the most severe pain score between groups but lower nonmenstrual pain trajectories and higher responder rates with E4/DRSP than with EE/DRSP (32). Open-label phase 3 analyses of premenstrual and menstrual symptoms in Europe and North America reported improvements mainly among starters, with reductions in pain, water retention, and negative affect, whereas switchers showed less consistent benefit (33). In adolescents, dysmenorrhea decreased by 34.8%, median pain score fell from 5.0 to 3.7, and pain medication use declined from 63.9% to 31.6% by cycle 6 (18).

### Certainty of the evidence

The certainty of the evidence by outcome, assessed using the GRADE framework, is summarized in **Table 3**.

Overall, the certainty of evidence was moderate for contraceptive efficacy, cycle control, common adverse events, and surrogate hemostatic and endocrine–metabolic outcomes. These ratings were primarily limited by study design, including open-label and noncomparative phase 3 studies, although consistency and precision across studies supported moderate certainty.

The certainty of evidence was high for dysmenorrhea reduction compared with placebo, based on a randomized, double-blind controlled trial with consistent and precise estimates.

Evidence for noncontraceptive outcomes such as endometriosis-related pain and menstrual symptoms was rated as low certainty, mainly due to open-label designs, small sample sizes, and indirectness.

The certainty of evidence for venous thromboembolism was very low, reflecting the rarity of events, limited statistical power of available studies, and reliance on noncomparative data.

Across outcomes, the main factors leading to rating down were risk of bias, indirectness of surrogate endpoints, and imprecision for rare clinical events, particularly in the context of thromboembolic safety.

**Figure 3:**
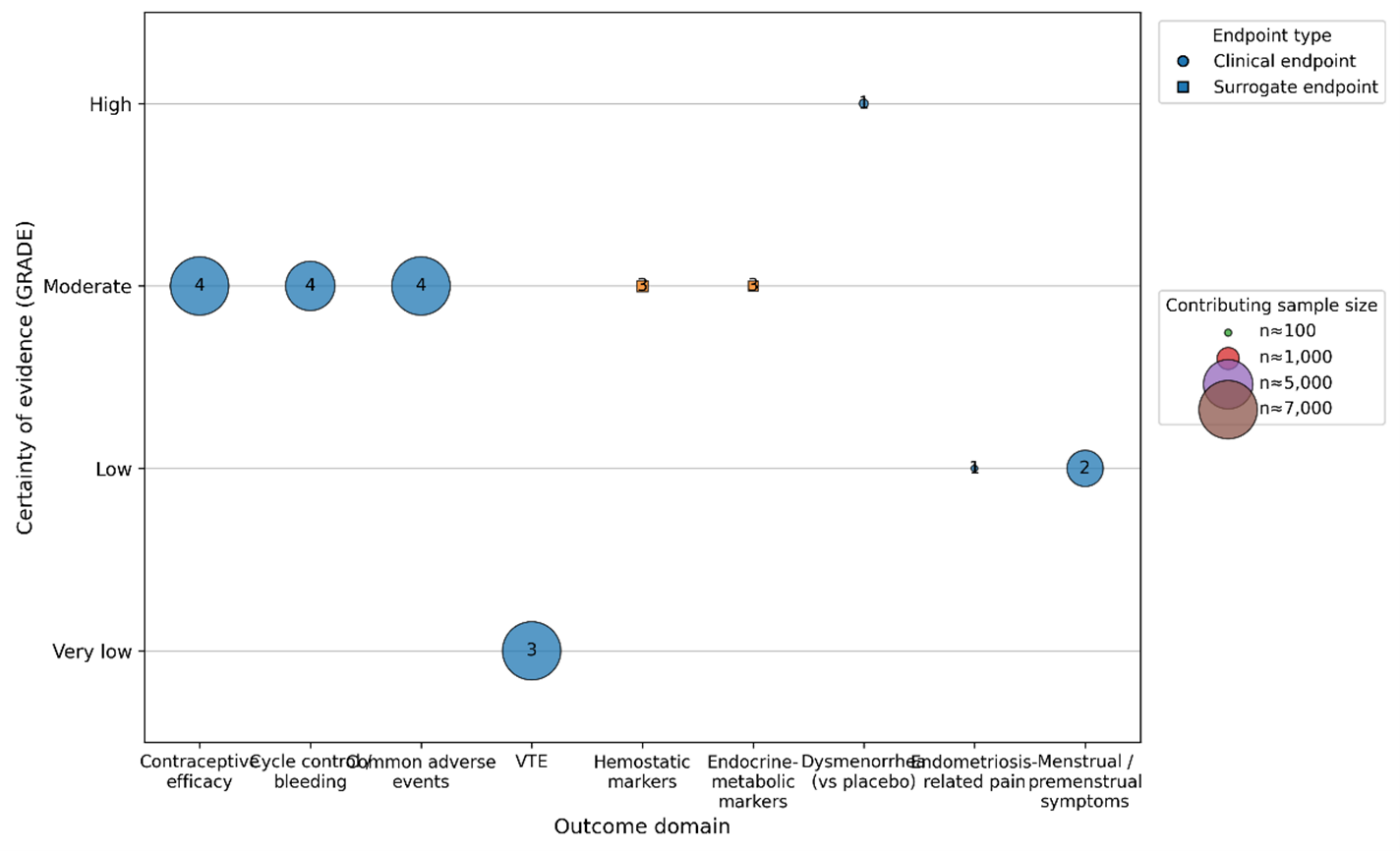
Evidence map of estetrol/drospirenone (E4/DRSP) across outcome domains. The vertical axis represents certainty of evidence according to the GRADE framework. Bubble size reflects the approximate contributing sample size for each outcome domain, and the number within each bubble indicates the number of contributing study reports. Circular markers denote clinical endpoints, whereas square markers denote surrogate endpoints. Sample sizes reflect contributing evidence bases and may include overlapping populations across related reports from the same phase 3 clinical development program.

The distribution of evidence across outcome domains, integrating certainty of evidence, contributing sample size, number of study reports, and endpoint type, is summarized in **Figure 3**.

## Discussion

This systematic review indicates that estetrol 15 mg/drospirenone 3 mg (E4/DRSP) provides a coherent clinical profile characterized by robust contraceptive efficacy, predictable cycle control, and acceptable tolerability, with the strongest evidence derived from the phase 3 contraceptive program and the highest-certainty noncontraceptive evidence derived from the placebo-controlled dysmenorrhea trial. Across outcome domains, the body of evidence was most consistent for efficacy, bleeding control, common adverse events, and biologic hemostatic/metabolic effects, whereas certainty was substantially lower for clinical thromboembolic safety and for some symptom-based secondary outcomes. This pattern reflects the structure of the available evidence base, which combines pivotal open-label contraceptive studies with mechanistic comparator trials and indication-specific studies. The evidence map (**Figure 3**) further illustrates this distribution, highlighting the concentration of moderate-certainty evidence in efficacy, cycle control, and tolerability, and the relative scarcity of high-certainty data for clinical safety outcomes.

The contraceptive findings are clinically relevant. In the phase 3 studies, E4/DRSP was associated with low pregnancy rates and stable effectiveness across major subgroups, while secondary analyses identified adherence as a key determinant of contraceptive failure. The consistency of efficacy signals across geographically distinct development programs supports the conclusion that the approved 24/4 regimen performs as an effective combined oral contraceptive under trial conditions. The certainty of evidence was rated as moderate rather than high, primarily because the pivotal studies were open-label and several supportive analyses were derived from the same clinical development program rather than independent confirmatory trials (6).

Cycle control is a major determinant of continuation and user satisfaction with combined hormonal contraception, and the present review suggests that E4/DRSP performs favorably in this domain. Across phase 3 and supportive studies, scheduled bleeding was generally predictable and unscheduled bleeding declined over time, including in adolescent users. These findings are consistent with the broader contraceptive literature, in which early-cycle irregular bleeding is common and adherence plays a critical role in cycle control and continuation (1). The observed influence of adherence on bleeding patterns in E4/DRSP studies aligns with established clinical experience rather than representing a product-specific limitation (1).

The safety and tolerability profile observed in this review was also broadly in line with expectations for a combined oral contraceptive (1, 2). The most frequently reported adverse events included headache, abnormal uterine bleeding, acne, breast tenderness, and mood-related symptoms, with relatively low rates of treatment discontinuation due to adverse events. These findings support the interpretation that E4/DRSP has an acceptable and clinically manageable tolerability profile within the context of combined hormonal contraception, although this should not be interpreted as absence of clinically relevant side effects (7, 8). Patient-reported outcomes are clinically relevant because satisfaction, well-being, bleeding acceptability, and sexual function may influence adherence and continuation. Available data suggest potentially favorable effects of E4/DRSP on satisfaction and well-being (17); however, robust evidence on sexual function remains limited. Data on libido, arousal, lubrication, dyspareunia, orgasmic function, sexual satisfaction, and sexual distress are scarce and should be addressed in future studies using validated instruments.

A major strength of the current evidence base is the consistency of the mechanistic hemostatic signal. Across randomized comparator studies, E4/DRSP had a smaller effect on thrombin generation, activated protein C resistance, D-dimer, SHBG, angiotensinogen, and several endocrine-metabolic markers than EE-containing comparators (20, 21, 23, 24). In women with polycystic ovary syndrome, metabolic effects on glucose tolerance and insulin-related indices were not meaningfully worse than those observed with EE/DRSP (25). These findings support the interpretation that E4/DRSP has a more limited hepatic and hemostatic impact than traditional EE-based contraceptives (20, 21, 23, 24). Classical hemostatic markers, including D-dimer, prothrombin fragment 1 + 2, protein S, antithrombin, SHBG, and angiotensinogen, provide useful information on estrogen-related hepatic and coagulation effects (20, 21, 23). However, global functional assays such as thrombin generation and APC resistance-based thrombin generation are particularly informative because they integrate the net balance between procoagulant and anticoagulant pathways (23, 24). The smaller effect of E4/DRSP on these integrated assays therefore provides a stronger mechanistic rationale for a potentially lower estrogen-induced procoagulant phenotype than that observed with EE-containing formulations (20, 21, 23, 24).

A further integrative layer is provided by a recently published nAPCsr-based predictive model linking ETP-based activated protein C resistance measurements to epidemiologically derived VTE relative risks across COC formulations (35). This model estimated a low relative VTE risk for E4/DRSP, close to that predicted for E2/NOMAC and substantially below estimates for several EE-containing formulations (35). Although such modelling cannot replace prospective postauthorization comparative epidemiology, it provides a quantitative bridge between functional coagulation assays and population-level thrombotic risk estimation (35). In this context, the lower nAPCsr and thrombin-generation impact observed with E4/DRSP should be interpreted not merely as isolated surrogate findings, but as part of a biologically coherent translational signal (23, 24, 35).

The key interpretive challenge is how far these biologic findings can be extrapolated to clinical vascular safety. Although the surrogate profile of E4/DRSP is favorable, this review does not establish a reduced risk of venous thromboembolism (VTE) compared with EE-containing combined oral contraceptives. The number of observed thromboembolic events in the clinical development program was very small, and the available studies were neither designed nor powered to estimate comparative VTE risk with precision. This limitation is particularly relevant given that differences in thrombotic risk between contraceptive formulations often require very large populations and real-world data to be reliably detected (1, 2, 4). Recent reviews and comparative epidemiologic studies have suggested that natural-estrogen–based combined contraceptives may be associated with a lower thrombotic risk than EE-containing formulations, with observational data for estradiol valerate/dienogest pointing in that direction (11).

In this context, emerging postmarketing evidence provides supportive but indirect signals. Disproportionality analyses from large pharmacovigilance databases, including EudraVigilance and FAERS, have reported lower proportionality reporting rates for thrombotic events with E4/DRSP than with EE-containing formulations, with values comparable to progestin-only regimens (36, 37). However, these analyses are based on spontaneous reporting systems and are inherently subject to underreporting, reporting bias, lack of reliable denominators, and residual confounding (36, 37). Accordingly, they should be interpreted as hypothesis-generating rather than confirmatory evidence of comparative safety.

In addition, real-world safety is currently being evaluated in a large international active surveillance postauthorization study, designed to assess the risk of venous and arterial thromboembolism associated with E4/DRSP in routine clinical practice (38). Such studies are expected to provide more robust comparative estimates of vascular risk, particularly for rare outcomes that cannot be adequately assessed within clinical development programs.

E4/DRSP should also be interpreted within the broader pharmacological transition from EE-based combined oral contraceptives toward formulations containing natural or body-identical estrogens (4, 11). Estradiol-based COCs, including E2V/DNG and E2/NOMAC, provide an important comparator framework because their development has similarly been driven by the aim of preserving contraceptive efficacy while reducing hepatic and hemostatic estrogenic stimulation (4, 11). For E2-containing COCs, the evidence base is more mature, with available postauthorization epidemiological data suggesting lower venous and arterial thromboembolic risk than conventional EE-containing formulations (11). By contrast, the evidence for E4/DRSP currently relies more heavily on randomized hemostatic biomarker studies, thrombin-generation data, clinical development safety observations, pharmacovigilance disproportionality analyses, and ongoing active-surveillance evaluation (7, 9, 10, 20, 21, 23, 24, 35–38). Although the maturity of evidence differs between E2- and E4-containing COCs, the direction of findings is consistent across natural-estrogen–based formulations and supports the biological plausibility of a lower thrombotic impact than that observed with EE-containing COCs (11, 20, 21, 36–38).

Taken together, the convergence of randomized hemostasis studies, thrombin-generation data, clinical development safety observations, pharmacovigilance analyses, evidence from natural estrogen-based contraception, and nAPCsr-based predictive modelling supports a biologically coherent and clinically plausible favorable thrombotic profile for E4/DRSP compared with EE-containing COCs (11, 20, 21, 23, 24, 35–38). However, definitive comparative clinical confirmation requires large active-comparator postauthorization studies.

Breast safety should be interpreted separately from breast tolerability. Available E4/DRSP trials were not designed or powered to evaluate breast cancer risk, and follow-up duration is insufficient to assess long-term breast oncological safety (7, 9, 19). Breast tenderness was reported as an adverse event and appeared clinically manageable, but this should not be interpreted as evidence of breast cancer safety (19). Long-term observational surveillance is required.

Evidence on bone health remains insufficient, particularly in adolescents and young users, for whom peak bone mass acquisition is clinically relevant (18, 25). Future studies should include bone mineral density, bone turnover markers, vitamin D status, IGF-1 axis parameters, and stratification by pubertal stage, menstrual maturity, and baseline bone health.

The noncontraceptive findings require differentiated interpretation. The strongest evidence was observed for dysmenorrhea, supported by a randomized, double-blind, placebo-controlled trial demonstrating clinically meaningful improvements in pain and quality-of-life outcomes (30). That study justifiably received the highest certainty rating in the review. By contrast, evidence for endometriosis-associated pain, adenomyosis-related symptoms, and premenstrual or menstrual symptom improvement was more heterogeneous and methodologically weaker. The endometriosis study was active-controlled but open-label (32), the adenomyosis analysis was *post hoc* (31), and the large symptom studies were based on open-label phase 3 populations in which improvements were more evident among starters than switchers. These findings are clinically promising but should be interpreted as supportive rather than definitive evidence of broader therapeutic benefit.

Several methodological considerations influence the interpretation of this review. First, multiple publications originated from the same phase 3 clinical development program, which may lead to overrepresentation of specific datasets if not carefully accounted for. Second, most pivotal studies were open-label, which has limited impact on objective outcomes such as pregnancy but may influence bleeding patterns, adherence reporting, and subjective symptom outcomes. Third, mechanistic studies were generally randomized but small and relied on surrogate endpoints, introducing indirectness in relation to clinical outcomes. The use of domain-specific risk-of-bias assessment combined with outcome-level GRADE evaluation was therefore essential to appropriately characterize the certainty of evidence.

This review also has limitations. The search strategy was restricted to major databases and published reports and did not include individual patient data, regulatory submissions, or unpublished postmarketing studies. No quantitative meta-analysis was performed because of heterogeneity in study design, populations, comparators, and endpoints, as well as partial overlap across study reports. In addition, Embase was not included in the formal database search, which represents a limitation, particularly for a pharmacological and contraceptive safety review. Reporting bias could not be formally assessed in the absence of a quantitative meta-analysis. Pharmacovigilance analyses were not included in the formal synthesis or GRADE assessment and were considered only as contextual external evidence. Finally, the evidence base remains largely derived from industry-sponsored clinical development programs, underscoring the need for independent real-world validation.

From a clinical perspective, E4/DRSP represents a relevant addition to the range of combined oral contraceptive options. It may be particularly suitable for women in whom contraceptive efficacy, cycle predictability, and a potentially lower hepatic and hemostatic impact are key considerations. However, clinical decision-making should remain grounded in established eligibility criteria for combined hormonal contraception, including individual risk assessment for venous thromboembolism, rather than assumptions of proven thromboembolic superiority. The most important next step for the field is the generation of robust comparative real-world evidence to clarify the vascular safety profile of E4/DRSP relative to established contraceptive options.

## Conclusions

Estetrol 15 mg/drospirenone 3 mg (E4/DRSP) is an effective combined oral contraceptive with predictable bleeding patterns and acceptable tolerability. Available evidence also supports a less pronounced hemostatic and endocrine–metabolic impact than that observed with ethinyl estradiol–containing comparators, although this advantage is currently supported mainly by surrogate biomarkers.

Certainty of evidence is moderate for efficacy, cycle control, common adverse events, and surrogate biologic outcomes, but very low for clinical thromboembolic risk. E4/DRSP therefore represents a clinically relevant addition to combined oral contraception. Nevertheless, the convergence of randomized hemostasis studies, thrombin-generation data, clinical development safety observations, pharmacovigilance analyses, evidence from natural estrogen-based contraception, and nAPCsr-based predictive modeling supports a biologically coherent and clinically plausible favorable thrombotic profile compared with EE-containing COCs. Definitive conclusions regarding vascular safety require dedicated comparative postauthorization studies.

## Data Availability

The original contributions presented in the study are included in the article/**Supplementary Material**. Further inquiries can be directed to the corresponding author.
