# Supplementary material for: Estetrol/drospirenone for combined oral contraception: a systematic review of efficacy, cycle control, and safety
Source: Front Endocrinol (Lausanne). 2026 Jul 24;17:1867343. doi: 10.3389/fendo.2026.1867343 (PMC13447189; doi:10.3389/fendo.2026.1867343)
Supplement: Supplementary file 1 [file DataSheet1.pdf]

Supplementary Material 1 - Full electronic search strategies

The literature search was conducted in PubMed/MEDLINE, Scopus, and Google Scholar to identify studies evaluating estetrol 15 mg/drospirenone 3 mg (E4/DRSP) as a combined oral contraceptive. Searches included studies published up to February 2026. No language restriction was applied at the search stage; eligibility was determined during screening. Reference lists of relevant publications were manually screened to identify additional eligible reports. Only full-text publications reporting original human data were considered.

1. PubMed/MEDLINE

Date of last search: February 2026
Search string: ("estetrol"[Title/Abstract] OR estetrol[MeSH Terms] OR estetrol[All Fields]) AND ("drospirenone"[Title/Abstract] OR drospirenone[MeSH Terms] OR drospirenone[All Fields]) AND ("contraception"[Title/Abstract] OR contraception[MeSH Terms] OR contracept*[Title/Abstract] OR "combined oral contraceptive*"[Title/Abstract] OR COC[Title/Abstract] OR COCs[Title/Abstract])

2. Scopus

Date of last search: February 2026
Search string:

TITLE-ABS-KEY (estetrol)

AND TITLE-ABS-KEY (drospirenone)

AND TITLE-ABS-KEY (contraception OR contracept* OR "combined oral contraceptive*" OR coc OR cocs)

3. Google Scholar

Date of last search: February 2026
Search string:

allintitle: estetrol drospirenone contraception

Additional Google Scholar search:

"estetrol" AND "drospirenone" AND contraception

4. Manual search

Reference lists of all eligible publications and relevant review articles were screened manually to identify additional reports not captured through database searching.

5. Eligibility applied after retrieval

Reports were eligible if they:

- evaluated **E4/DRSP**, preferably the marketed **15 mg/3 mg 24/4 regimen**;
- included women of reproductive age or adolescent users in clinically relevant contraceptive settings;
- reported outcomes related to contraceptive efficacy, bleeding/cycle control, safety, tolerability, ovarian suppression, hemostatic or metabolic effects, or noncontraceptive clinical outcomes; and
- used an interventional or comparative design, including randomized trials, nonrandomized comparative studies, pooled analyses, and prespecified or post hoc analyses.

Reports were excluded if they were narrative reviews, editorials, letters without original data, conference abstracts without sufficient methodological detail, duplicate publications without additional outcome data, or studies evaluating nonrelevant formulations not considered clinically informative for interpretation of the approved E4/DRSP regimen.
