# Supplementary material for: Estetrol/drospirenone for combined oral contraception: a systematic review of efficacy, cycle control, and safety
Source: Front Endocrinol (Lausanne). 2026 Jul 24;17:1867343. doi: 10.3389/fendo.2026.1867343 (PMC13447189; doi:10.3389/fendo.2026.1867343)
Supplement: Supplementary file 2 [file DataSheet2.pdf]

Supplementary Material 2. PRISMA 2020 Checklist

Section and topic	Item no.	PRISMA 2020 checklist item	Location where item is reported
**Title**	1	Identify the report as a systematic review.	Title page: "A Systematic Review of Efficacy, Cycle Control, and Safety."
**Abstract**	2	See the PRISMA 2020 for Abstracts checklist.	Abstract: Objective, Methods, Results, Conclusions. The abstract summarizes review objective, eligibility, databases, included studies, main findings, certainty of evidence, and conclusions.
**Introduction: Rationale**	3	Describe the rationale for the review in the context of existing knowledge.	Introduction, paragraphs 1–4. The rationale is based on the need to synthesize heterogeneous evidence on E4/DRSP efficacy, cycle control, tolerability, hemostatic/endocrine-metabolic effects, thromboembolic safety, and noncontraceptive outcomes.
**Introduction: Objectives**	4	Provide an explicit statement of the objective(s) or question(s) the review addresses.	End of Introduction. The objective was to synthesize evidence on contraceptive efficacy, cycle control, safety, and selected noncontraceptive effects of E4 15 mg/DRSP 3 mg, with outcome-level GRADE assessment.
**Methods: Eligibility criteria**	5	Specify the inclusion and exclusion criteria for the review and how studies were grouped for the syntheses.	Methods, "Eligibility criteria"; Table 1. PICO framework. Eligible reports included interventional or comparative human studies evaluating E4/DRSP and reporting relevant clinical, safety, tolerability, physiologic, hemostatic, metabolic, or noncontraceptive outcomes. Exclusion criteria included reviews, expert opinions, editorials, letters without original data, conference abstracts without sufficient methodological detail, and duplicate reports without additional outcome

			data. Studies were grouped by prespecified outcome domains.
**Methods: Information sources**	6	Specify all databases, registers, websites, organizations, reference lists, and other sources searched or consulted to identify studies. Specify the date when each source was last searched or consulted.	Methods, “Information sources and search strategy”; Supplementary Material 1. PubMed/MEDLINE, Scopus, Google Scholar, and manual reference screening were used. Searches included articles published through February 2026. Additional postmarketing pharmacovigilance, predictive-modeling, and registry sources published or updated after the formal search date were cited only as contextual evidence in the Discussion and were not included in the formal synthesis or GRADE assessment.
**Methods: Search strategy**	7	Present the full search strategies for all databases, registers, and websites, including filters and limits used.	Methods, “Information sources and search strategy”; Supplementary Material 1. Core terms included “estetrol”, “drospirenone”, and “contraception”, used alone and in combination. Full electronic search strategies are provided in Supplementary Material 1.
**Methods: Selection process**	8	Specify the methods used to decide whether a study met the inclusion criteria, including how many reviewers screened records and reports, whether they worked independently, and details of automation tools.	Methods, “Study selection.” Two reviewers independently screened titles/abstracts and full texts. Discrepancies were resolved by discussion and consensus. No automation tools were used in screening.
**Methods: Data collection process**	9	Specify the methods used to collect data from reports, including how many reviewers collected data, whether they worked independently, processes for obtaining or	Methods, “Data extraction.” Two reviewers independently extracted data using a standardized form. Discrepancies were resolved by consensus. No automation tools were used.

		confirming data, and details of automation tools.	
**Methods: Data items**	10a	List and define all outcomes for which data were sought. Specify whether all results compatible with each outcome domain were sought.	Methods, “Data extraction.” Outcomes were grouped into contraceptive efficacy, bleeding/cycle control, safety and tolerability, physiologic and mechanistic endpoints, and noncontraceptive clinical outcomes.
**Methods: Data items**	10b	List and define all other variables for which data were sought. Describe assumptions made about missing or unclear information.	Methods, “Data extraction”; Table 2. Extracted variables included first author, year, geographic setting, study period when available, sample size, population characteristics, design, comparator, treatment duration/follow-up, objective, and main outcomes. Missing or unclear information was reported as not stated when unavailable.
**Methods: Study risk-of-bias assessment**	11	Specify the methods used to assess risk of bias in included studies, including details of tools, number of reviewers, independence, and processes for resolving disagreements.	Methods, “Risk-of-bias assessment”; Results, “Risk of bias across included reports”; Figure 2. Randomized trials were assessed using RoB 2. Nonrandomized comparative studies were assessed according to ROBINS-I principles. Single-arm, pooled, post hoc, and subgroup analyses were evaluated using design-adapted criteria. Two reviewers independently performed assessments, with disagreements resolved by consensus.
**Methods: Effect measures**	12	Specify for each outcome the effect measures used in the synthesis or presentation of results.	Methods, “Data synthesis”; Results by outcome domain. Extracted measures included Pearl Index, life-table pregnancy rates, adverse-event rates, discontinuation rates, absolute event rates, between-group differences, confidence intervals, biomarker changes, and symptom-score changes as reported by original studies.

**Methods: Synthesis methods**	13a	Describe the processes used to decide which studies were eligible for each synthesis.	Methods, "Eligibility criteria," "Data extraction," and "Data synthesis." Studies were assigned to prespecified outcome domains according to their primary objective and reported outcomes.
**Methods: Synthesis methods**	13b	Describe any methods required to prepare the data for presentation or synthesis, such as handling missing summary statistics or data conversions.	Methods, "Data synthesis." Data were extracted and summarized descriptively as reported in the original publications. No data conversions or imputation procedures were performed.
**Methods: Synthesis methods**	13c	Describe any methods used to tabulate or visually display results of individual studies and syntheses.	Methods, "Presentation of results"; Results; Table 2; Table 3; Figure 3. Study characteristics were tabulated in Table 2. Outcome-level certainty was summarized in Table 3. An evidence map was constructed to integrate outcome domains, certainty of evidence, contributing sample size, number of reports, and endpoint type.
**Methods: Synthesis methods**	13d	Describe any methods used to synthesize results and provide rationale for choices. If meta-analysis was performed, describe models and methods.	Methods, "Data synthesis." A qualitative systematic synthesis was performed because of heterogeneity in study design, populations, comparators, follow-up duration, and endpoint definitions, with partial overlap across related reports. No quantitative meta-analysis was performed.
**Methods: Synthesis methods**	13e	Describe any methods used to explore possible causes of heterogeneity among study results.	Methods, "Data synthesis"; Discussion. Heterogeneity was explored narratively by grouping studies according to outcome domain, study design, comparator, population, and endpoint type.
**Methods: Synthesis methods**	13f	Describe any sensitivity analyses conducted to assess robustness of synthesized results.	Not applicable. No quantitative meta-analysis or formal sensitivity analyses were performed because of substantial heterogeneity and partial overlap across related study reports.

**Methods: Reporting bias assessment**	14	Describe any methods used to assess risk of bias due to missing results in a synthesis arising from reporting biases.	Methods, "Risk-of-bias assessment" and "Data synthesis"; Table 3. Reporting bias/publication bias was not formally assessed because no quantitative meta-analysis was performed and the evidence base was heterogeneous in design, outcome definitions, comparators, populations, and partially overlapping study reports.
**Methods: Certainty assessment**	15	Describe any methods used to assess certainty or confidence in the body of evidence for an outcome.	Methods, "Certainty of the evidence"; Table 3. Certainty was assessed by outcome using the GRADE framework, considering risk of bias, inconsistency, indirectness, imprecision, and publication bias.
**Results: Study selection**	16a	Describe the results of the search and selection process, from number of records identified to number of studies included, ideally using a flow diagram.	Results, "Study selection"; Figure 1. PRISMA 2020 flow diagram. A total of 312 records were identified; 74 duplicates were removed; 238 records were screened; 170 were excluded; 68 reports were sought for retrieval; 3 were not retrieved; 65 full-text reports were assessed; 40 were excluded; and 25 publications/study reports were included.
**Results: Study selection**	16b	Cite studies that appeared to meet inclusion criteria but were excluded, and explain why.	Results, "Study selection"; Figure 1. Excluded full-text reports were categorized by predefined reasons, including lack of relevant clinical data, absence of original data, non-relevant intervention, and insufficient methodological detail.
**Results: Study characteristics**	17	Cite each included study and present its characteristics.	Results, "Study characteristics"; Table 2. The 25 included publications/study reports are summarized by author, year, country/region, study design, population, sample size, comparator, duration, primary objective, and outcome domain.
**Results: Risk of bias in studies**	18	Present assessments of risk of bias for each included study.	Results, "Risk of bias across included reports"; Figure 2. Risk-of-bias assessments are summarized across included reports and

			incorporated into the GRADE judgments in Table 3.
**Results: Results of individual studies**	19	For all outcomes, present summary statistics for each study and, where applicable, effect estimates and precision.	Results by outcome domain. Individual study findings are presented for contraceptive efficacy, bleeding/cycle control, safety and tolerability, VTE/cardiovascular safety, hemostatic and endocrine-metabolic effects, ovarian suppression, and noncontraceptive clinical outcomes.
**Results: Results of syntheses**	20a	For each synthesis, briefly summarize the characteristics and risk of bias among contributing studies.	Results, “Risk of bias across included reports,” and outcome-specific Results sections. Characteristics are summarized in Table 2 and risk-of-bias judgments in Figure 2.
**Results: Results of syntheses**	20b	Present results of all statistical syntheses conducted. If meta-analysis was done, present summary estimate and precision.	Not applicable. No quantitative meta-analysis was performed. Results were synthesized narratively and descriptively.
**Results: Results of syntheses**	20c	Present results of investigations of possible causes of heterogeneity among study results.	Results and Discussion. Heterogeneity was interpreted narratively according to study design, comparator, endpoint type, population, and overlap among related study reports.
**Results: Results of syntheses**	20d	Present results of all sensitivity analyses conducted to assess robustness of synthesized results.	Not applicable. No formal sensitivity analyses were performed because no quantitative meta-analysis was conducted.
**Results: Reporting biases**	21	Present assessments of risk of bias due to missing results arising from reporting biases.	Methods, “Data synthesis”; Results, Table 3; Discussion, limitations. Reporting bias/publication bias was not formally assessed due to absence of quantitative meta-analysis, heterogeneity, and partial overlap across reports.

**Results: Certainty of evidence**	22	Present assessments of certainty or confidence in the body of evidence for each outcome.	Results, “Certainty of the evidence”; Table 3; Figure 3. Certainty was moderate for contraceptive efficacy, cycle control, common adverse events, and surrogate hemostatic/endocrine-metabolic outcomes; high for dysmenorrhea versus placebo; low for endometriosis-related and menstrual/premenstrual symptom outcomes; and very low for clinical thromboembolic risk.
**Discussion: Discussion**	23a	Provide a general interpretation of the results in the context of other evidence.	Discussion, paragraphs 1–11. Findings are interpreted in relation to established combined hormonal contraception, EE-containing comparators, natural estrogen-based COCs, pharmacovigilance evidence, nAPCsr-based predictive modelling, and ongoing postauthorization surveillance.
**Discussion: Discussion**	23b	Discuss limitations of the evidence included in the review.	Discussion, limitations paragraphs. Limitations include open-label and single-arm designs, overlapping study populations, small mechanistic studies, surrogate endpoints, limited VTE event numbers, industry-sponsored development programs, limited long-term data, and insufficient evidence for breast cancer risk, bone health, and sexual function.
**Discussion: Discussion**	23c	Discuss limitations of the review processes used.	Discussion, limitations paragraph. Limitations include no quantitative meta-analysis, no formal reporting/publication bias assessment, no individual patient data, no regulatory submissions or unpublished postmarketing studies, and absence of Embase from the formal database search.
**Discussion: Discussion**	23d	Discuss implications of the results for practice, policy, and future research.	Discussion, final paragraphs; Conclusions. E4/DRSP is interpreted as a clinically relevant contraceptive option with favorable cycle control and biologically plausible lower hepatic/hemostatic impact than EE-

			containing formulations, while definitive comparative vascular safety requires active-comparator postauthorization studies.
**Other information: Registration and protocol**	24a	Provide registration information for the review, including register name and registration number, or state that the review was not registered.	Methods, "Study design and reporting framework." The protocol was not prospectively registered. Eligibility criteria and the review framework were defined a priori.
**Other information: Registration and protocol**	24b	Indicate where the review protocol can be accessed, or state that a protocol was not prepared.	Methods, "Study design and reporting framework." A protocol was not prospectively registered or made publicly available.
**Other information: Registration and protocol**	24c	Describe and explain any amendments to information provided at registration or in the protocol.	Not applicable. The review was not prospectively registered.
**Other information: Support**	25	Describe sources of financial or non-financial support for the review and the role of the funders or sponsors.	Funding statement. "The authors received no specific funding for this work."
**Other information: Competing interests**	26	Declare any competing interests of review authors.	Conflicts of interest statement. "The authors declare no competing interests related to this work."
**Other information: Availability of data, code, and other materials**	27	Report which data, code, and other materials are publicly available and where they can be found.	Data availability statement. All data relevant to the study are included in the article and supplementary materials. Additional information can be provided by the corresponding author upon reasonable request.
